# Supplementary material for: A Critical View of the Application of the APEX Software (Aqueous Photochemistry of Environmentally-Occurring Xenobiotics) to Predict Photoreaction Kinetics in Surface Freshwaters
Source: Molecules. 2019 Dec 18;25(1):9. doi: 10.3390/molecules25010009 (PMC7017383; doi:10.3390/molecules25010009)
Supplement: Supplementary file 1 [file molecules-25-00009-s001.zip › 3.2.4_gcc-4.4.0.zip!/3.2.4_gcc-4.4.0/3.2.4_gcc-4.4.0/doc/octave/html/interpreter/How-You-Can-Contribute-to-Octave.html]

How You Can Contribute to Octave - Untitled


Next: Distribution,
Previous: Acknowledgements,
Up: Preface


---

### How You Can Contribute to Octave

There are a number of ways that you can contribute to help make Octave a
better system. Perhaps the most important way to contribute is to write
high-quality code for solving new problems, and to make your code freely
available for others to use. See Contributing Guidelines, for detailed
information on contributing new code.

If you find Octave useful, consider providing additional funding to
continue its development. Even a modest amount of additional funding
could make a significant difference in the amount of time that is
available for development and support.

If you cannot provide funding or contribute code, you can still help
make Octave better and more reliable by reporting any bugs you find and
by offering suggestions for ways to improve Octave. See Trouble, for
tips on how to write useful bug reports.
